# When Sound Fades: Depression and Anxiety in Adults with Hearing Loss—A Cross-Sectional Study

**DOI:** 10.3390/healthcare13243320

**Published:** 2025-12-18

**Authors:** Serkan Dedeoglu, Serdar Ferit Toprak, Enes Sırma, Süleyman Dönmezdil

**Affiliations:** 1 Department of Otorhinolaryngology, University of Health Sciences Gazi Yasargil Training and Research Hospital, Diyarbakır 21010, Turkey; enessirmamd@gmail.com; 2 Department of Audiology, Artuklu University, Mardin 47300, Turkey; serdarferit@yahoo.com; 3 Department of Psychology, Artuklu University, Mardin 47300, Turkey; suleymandonmezdil@artuklu.edu.tr

**Keywords:** hearing loss, depression, anxiety, psychological impact, quality of life

## Abstract

**Background:** Hearing loss is a prevalent sensory impairment with substantial psychosocial consequences. This cross-sectional study investigated the relationship between audiometric hearing loss and mood disturbances in adults aged 18–65 years who reported hearing difficulties for at least six months. **Methods:** Objective hearing level was assessed using the better-ear pure-tone average (PTA), and subjective hearing handicap was measured with the Hearing Handicap Inventory for Adults (HHIA). Standardized mood assessments included the Beck Depression Inventory-II (BDI-II) and the Beck Anxiety Inventory (BAI). **Results:** The study found that higher HHIA scores, indicating greater perceived hearing handicap, were strongly correlated with more severe depression and anxiety (ρ ≈ 0.45 and 0.38, respectively; *p* < 0.001). In contrast, objective PTA showed weaker associations with mood scores. Regression analyses, adjusted for age and gender, confirmed that perceived hearing handicap (HHIA) was the strongest independent predictor of both depression (standardized β ≈ 0.37, *p* < 0.001) and anxiety (β ≈ 0.33, *p* < 0.01), accounting for about 30% of the variance in mood scores. Nearly one-third of participants had clinically significant depression (BDI-II ≥ 20), which is substantially higher than community norms. The cross-sectional design and potential selection bias are limitations. **Conclusions:** Even mild-to-moderate hearing loss can result in significant depressive and anxious symptoms when individuals perceive themselves as handicapped. Early identification of hearing problems, routine psychosocial screening (e.g., a simple two-question survey), and integrated care are essential for improving quality of life.

## 1. Introduction

Hearing impairment is a major disability affecting millions globally. It causes communication problems, social isolation, loneliness, and a higher risk of mood disorders. In U.S. surveys, 11.4% of adults with hearing loss have moderate-to-severe depression with the risk in adults 40–55 is especially high. In this group, untreated hearing loss raises depression risk by over 200% [1]. Meta-analyses link hearing loss to a 1.3–1.4 times higher risk of depression and nearly double the risk for anxiety [2]. These effects may arise from social isolation, reduced engagement, and the neurobiological impact of hearing loss [3]. Recent reviews confirm that adults with hearing loss have much higher odds of depression [4].

Most evidence concerns older adults, but less is known about younger and middle-aged adults (18–65) with early hearing loss. We do not fully understand how mild, untreated hearing loss affects mood in this group. Some individuals with mild loss may still report significant mood problems if they perceive their condition as severe. This study explored whether hearing problems predict depression and anxiety in a clinical sample of adults under 65. We measured both objective hearing (PTA) and subjective handicap (HHIA). We hypothesized that greater hearing loss-especially if perceived as worse- would be associated with higher depression (BDI-II) and anxiety (BAI) scores. We also examined whether early diagnosis and treatment of hearing loss influences these mood outcomes.

Hearing difficulties can often lead to emotional challenges. Sensorineural hearing loss (SNHL) in older adults has been linked with higher depression and anxiety. Lawrence et al. (2020) found that older adults with hearing loss had more depression than those with normal hearing [4]. A recent review by Wei et al. (2024) confirmed that hearing loss raises depression risk (pooled OR ≈ 1.35) [5]. Hearing loss also appears to increase anxiety: Zhang et al. (2024) reported that around 40% of people with SNHL experience significant anxiety (OR ≈ 1.83 vs. the general population) [2]. Tinnitus, often accompanying hearing loss, is similarly associated with elevated psychiatric symptoms. These findings highlight the profound psychosocial impact of hearing disorders.

Several mechanisms may underlie the connection between hearing loss and mood. Communication difficulties can cause social isolation and loss of support, contributing to depression. Biological changes may also play a role: hearing loss can induce neuroplastic changes in the brain. Neuroimaging shows altered connections between auditory and emotional brain regions in individuals with hearing loss, aligning with observed mood symptoms. Animal studies suggest hearing loss can alter mood-related chemicals, such as serotonin [6]. Hearing difficulties can also heighten stress, compounding emotional strain. These changes support the idea that not just hearing loss itself, but feeling handicapped by hearing loss, raises the risk of mood issues.

Self-perceived handicap often affects outcomes more than measured hearing thresholds [7]. The Hearing Handicap Inventory (HHIA/HHIE) measures the extent to which hearing loss impacts daily life. Two people with similar hearing test results can experience very different handicaps depending on their coping skills and support [8]. Self-perceived handicap is a better predictor of quality of life and help-seeking than pure-tone thresholds [9]. Most research focuses on older adults; young and middle-aged groups are understudied. Recognizing these gaps underscores the importance of identifying mood effects early and considering early interventions.

In summary, this study examined the relationship between hearing loss and depression/anxiety in adults, using the HHIA to quantify perceived hearing difficulties and the Beck tools for mood. We adjusted for age, gender, and audiometric results to determine whether subjective handicap adds explanatory value for psychological distress. We expected that self-perceived hearing impairment would be linked to higher depression and anxiety. By focusing on adults under 65, this work addresses an understudied demographic. Our goal is to better understand the hearing loss–mental health relationship and to highlight opportunities for integrated audiological and psychosocial care.

## 2. Materials and Methods

### 2.1. Study Design and Participants

We conducted a cross-sectional study in an audiology clinic and via community outreach between August and October 2025. Adults aged 18–65 with at least 6 months of self-reported hearing trouble were eligible to participate. The sample size was determined a priori: expecting a moderate correlation (r ≈ 0.30) [10], with α = 0.05 (two-tailed) and power = 0.80, we required a minimum of 84 participants. We aimed to recruit 100 individuals to allow for potential dropouts.

### 2.2. Inclusion and Exclusion Criteria

Inclusion criteria were age 18–65 and a self-reported hearing difficulty persisting for ≥6 months (defining “subjective hearing loss” as answering “yes” to having hearing trouble or difficulty understanding speech). Exclusion criteria were congenital or childhood-onset deafness; any diagnosed psychiatric illness predating the onset of hearing loss; active substance abuse; neurological disease (e.g., stroke or dementia); and any active ear disease. We also excluded individuals for whom severe tinnitus was the primary complaint, those undergoing cancer treatment, or those with any severe medical illness known to affect mood. Use of antidepressant or anxiolytic medication was not an exclusion criterion if dosage had been stable for ≥3 months, but medication use was recorded. All participants provided written informed consent, and the study protocol was approved by the local institutional review board.

### 2.3. Audiometric Assessment

All participants underwent standard pure-tone audiometry administered by a certified audiologist in a soundproof booth. Air-conduction hearing thresholds were measured at octave frequencies from 0.5 through 8 kHz in each ear. The better-ear PTA (the average threshold at 0.5, 1, 2, 4 kHz in the better-hearing ear) was calculated as the measure of objective hearing level. Hearing loss severity was classified using conventional WHO criteria: normal (<25 dB HL), mild (25–40 dB HL), moderate (41–70 dB HL), or severe (>70 dB HL) based on the PTA.

### 2.4. Subjective Hearing Handicap

We assessed perceived hearing disability using the Hearing Handicap Inventory for Adults (HHIA), a 25-item self-report questionnaire with two subscales (13 emotional and 12 social/situational items) [11]. Each item is answered “Yes” (4 points), “Sometimes” (2 points), or “No” (0 points), yielding a total score from 0 to 100, with higher scores indicating a greater perceived hearing handicap. The HHIA has well-established reliability and validity. In our sample, the total HHIA demonstrated excellent internal consistency (Cronbach’s α = 0.89).

### 2.5. Psychological Measures

Depressive symptoms were measured with the Beck Depression Inventory-II (BDI-II), a 21-item self-report instrument evaluating depression severity over the past two weeks. Each item is rated 0–3; total scores range from 0 to 63, with higher scores reflecting more severe depression. We categorized BDI-II scores as minimal (0–13), mild (14–19), moderate (20–28), or severe (≥29) per standard guidelines [12]. Anxiety was measured with the Beck Anxiety Inventory (BAI), a 21-item self-report scale assessing common anxiety symptoms over the past week (each item 0–3; total 0–63). Established cutoffs for the BAI are minimal (0–7), mild (8–15), moderate (16–25), and severe (≥26) anxiety. Both the BDI-II and BAI have been used in hearing-impaired populations [13] and showed high internal consistency in our sample (Cronbach’s α = 0.91 for BDI-II; 0.90 for BAI).

### 2.6. Procedure

After providing informed consent, participants completed questionnaires on demographics and medical history, followed by the HHIA, BDI-II, and BAI in a quiet room. A researcher was available to clarify any questionnaire items if needed (for example, to assist with literacy or wording issues). Participants were assured that their responses on the psychological questionnaires were confidential and would not affect their clinical care or hearing test results. Following the surveys, pure-tone audiometry was performed. We recorded basic demographic information (age, sex, education level, employment status) and clinical factors such as duration of perceived hearing loss, whether the loss was unilateral or bilateral, and current use of hearing aids (yes/no). We also collected information on common comorbid medical conditions (diabetes, hypertension, thyroid disease), as these can co-occur with hearing loss and potentially affect mood.

### 2.7. Statistical Analysis

All analyses were conducted using SPSS Statistics version 27 (IBM Corp., Armonk, NY, USA). We first computed descriptive statistics for all variables. Continuous variables are presented as mean ± standard deviation (SD) if approximately normally distributed, or median (interquartile range) if markedly skewed. Categorical variables are summarized as frequencies and percentages. The distribution of key continuous measures (BDI-II, BAI, HHIA, PTA) was assessed using the Shapiro–Wilk test. BDI-II and HHIA scores were significantly right-skewed (Shapiro–Wilk *p* < 0.001 for each), whereas BAI and age were approximately normal (*p* > 0.05). Therefore, for group comparisons involving non-normally distributed variables, nonparametric tests were used. Specifically, we compared mean depression and anxiety scores between subgroups of interest using independent-samples *t*-tests (for roughly normal distributions) or the Mann–Whitney U test (for skewed distributions). We examined differences in mood scores by sex (male vs. female) and by hearing loss severity (mild vs. moderate-to-severe, using PTA > 40 dB HL to define moderate-to-severe).

Correlations between hearing loss metrics and psychological scores were analyzed using Pearson’s correlation for approximately normal variables and Spearman’s rank-order correlation for non-normal variables. We calculated bivariate correlations between HHIA scores and BDI-II, HHIA and BAI, and PTA (dB HL) and each mood scale. Correlation effect sizes were interpreted using standard benchmarks (small ~0.10, moderate ~0.30, large ≥0.50).

Finally, to identify independent predictors of mood outcomes, we performed multiple linear regression analyses for depression and anxiety scores. Separate models were constructed with BDI-II or BAI score as the dependent variable. Candidate predictors included perceived hearing handicap (HHIA score), objective hearing level (PTA in dB), age, and sex. These covariates were selected a priori based on their clinical or epidemiological relevance. Before running the regressions, we checked for multicollinearity; the correlation between HHIA and PTA was r = 0.36 (*p* < 0.001), indicating only moderate overlap (variance inflation factor ~1.15). We proceeded to include all predictors in each model (enter method). Regression assumptions were verified by inspecting residual plots for homoscedasticity and linearity, and by confirming normal distribution of residuals (Shapiro–Wilk *p* > 0.05 for both models). We report unstandardized coefficients, standardized β, 95% confidence intervals, *p*-values for each predictor, and the model R^2^ (adjusted) as a measure of explained variance. A two-tailed *p* < 0.05 was considered statistically significant.

## 3. Results

### 3.1. Sample Characteristics

We enrolled 100 participants (52% male) with a mean age of 45.3 ± 11.2 years. Based on audiometry, 62% had bilateral mild hearing loss (PTA 25–40 dB HL) and 38% had moderate-to-severe loss (PTA > 40 dB HL). Forty-one percent of participants reported current use of a hearing aid. Regarding education, 70% had completed at least a college degree. Seventy-eight percent were employed either full-time or part-time. Comorbid medical conditions were present in 34% of the sample (the most common being diabetes, hypertension, or thyroid disorders). Key clinical and demographic characteristics are summarized in Table 1.

Mean HHIA score was 42.6 (SD 22.8, range 0–92), indicating a substantial perceived handicap on average. The mean BDI-II score was 16.8 (SD 9.4) and the mean BAI score was 14.2 (SD 8.7). Based on conventional cutoffs, 53% of participants reported at least mild depressive symptoms (BDI-II > 13), and 28% met criteria for moderate-to-severe depression (BDI-II ≥ 20). For anxiety, 22% of participants had moderate-to-severe symptoms (BAI ≥ 16). As shown in Table 1, participants with moderate-to-severe hearing loss (PTA > 40 dB) had significantly higher median BDI-II scores than those with mild loss (median 21 vs. 13, Mann–Whitney U, *p* < 0.01). Their anxiety scores were also higher on average (median 15 vs. 10), though this difference did not reach statistical significance (*p* = 0.07). We observed no significant differences in depression or anxiety scores between male and female participants, nor between those aged≤ 45 vs. >45 years.

### 3.2. Correlation Analyses

**Figure 1:**
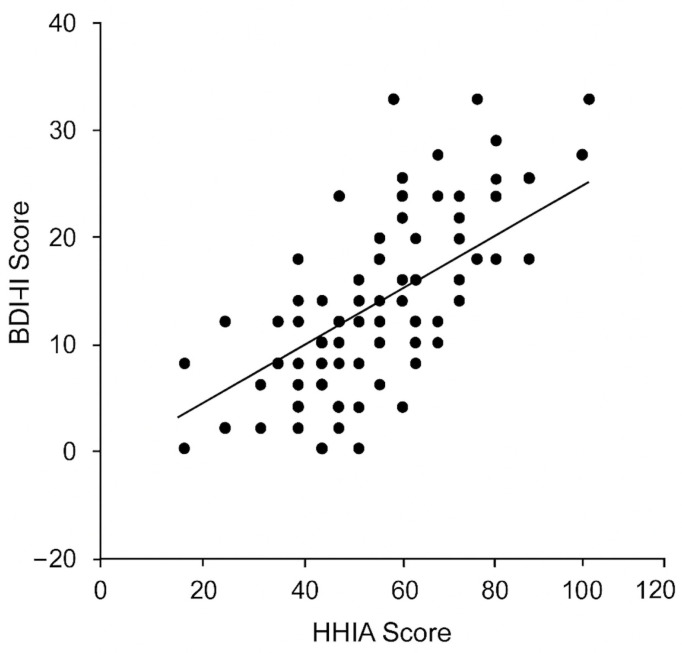
Scatterplot of perceived hearing handicap (HHIA score) vs. BDI-II depression score. Each point represents one participant; the black trend line indicates the positive correlation (Spearman ρ ≈ 0.45, *p* < 0.001).

**Figure 2:**
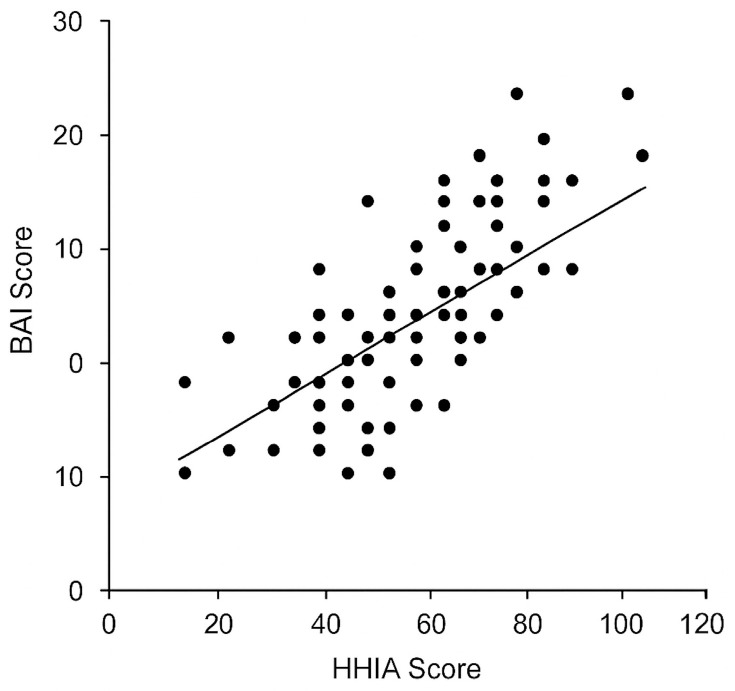
Scatterplot of perceived hearing handicap (HHIA score) vs. BAI anxiety score. A positive association is evident (ρ ≈ 0.38, *p* < 0.001).

HHIA scores were positively correlated with both depression and anxiety severity. Spearman’s ρ was +0.45 for HHIA vs. BDI-II (*p* < 0.001; see Figure 1) and +0.38 for HHIA vs. BAI (*p* < 0.001; Figure 2). By contrast, objective PTA (dB HL) was only weakly correlated with BDI-II (ρ = +0.22, *p* = 0.03) and was not significantly correlated with BAI (ρ = +0.18, *p* = 0.07). HHIA and PTA were moderately correlated with each other (ρ = +0.36, *p* < 0.001), indicating that poorer hearing tends to increase perceived handicap, though with considerable individual variability. As expected, BDI-II and BAI were strongly intercorrelated (r = +0.68, *p* < 0.001). The correlation matrix is shown in Table 2.

### 3.3. Regression Analyses

We conducted multivariate linear regressions to determine independent predictors of depression and anxiety (Table 3). For depression (BDI-II as outcome), the overall model was significant (F(4,95) = 12.1, *p* < 0.001, adjusted R^2^ ≈ 0.32). HHIA score emerged as the strongest independent predictor (standardized β = 0.37, 95% CI: 0.22 to 0.52, *p* < 0.001). This indicates that for every 10-point increase in HHIA, the BDI-II score rose by approximately 3.7 points, on average. PTA had a positive but non-significant effect (β = 0.12, *p* = 0.16), suggesting that the influence of objective hearing level on depression was largely mediated by the perceived handicap. Neither age nor sex was significantly related to BDI-II score in this model. For anxiety (BAI as outcome), a similar pattern was observed (F(4,95) = 8.5, *p* < 0.001, adjusted R^2^ ≈ 0.26). HHIA was again the only significant predictor (β = 0.33, 95% CI: 0.15 to 0.51, *p* = 0.001), whereas PTA, age, and sex all had *p* > 0.15. In summary, greater self-perceived hearing impairment was associated with higher depression and anxiety scores, even after accounting for objective hearing loss and demographics.

Notably, the regression findings support our hypothesis: greater perceived hearing difficulty is an independent risk factor for worse mood outcomes in this population.

## 4. Discussion

Our study demonstrates a robust association between hearing loss and mood disturbances in adults, in line with and extending prior research. Importantly, the subjective impact of hearing loss (as measured by HHIA) showed a substantially stronger relationship with psychological distress than the objective audiogram did. Participants who felt more handicapped by their hearing difficulties had much higher depression and anxiety scores, even after controlling for age, sex, and audiometric hearing level. This finding underscores that the psychosocial repercussions of hearing loss—rather than the audiometric severity alone—drive much of the mood impact. It is consistent with the notion that factors like social isolation, communication stress, and loss of autonomy mediate the link between hearing loss and depression. At the same time, we acknowledge the possibility of reverse causation: heightened depression or anxiety might itself inflate one’s perceived hearing difficulties. In other words, mood disturbances and perceived hearing handicap could potentially exacerbate each other. This bidirectional perspective warrants further investigation.

We observed strikingly elevated rates of mood symptoms in our sample. About 28% of participants had BDI-II scores in the moderate-to-severe range (≥20), which far exceeds the roughly 5–10% prevalence of depression in the general adult population [14]. Similarly, 22% of our sample had moderate or severe anxiety (BAI ≥ 16). These figures align with epidemiological reports that individuals with hearing loss have roughly twice the odds of depression compared to those without hearing impairment [1], and similarly higher anxiety prevalence (approximately 1.8-fold) [2,15]. Our findings reinforce the notion that hearing impairment carries a hidden mental health burden: even those with relatively mild hearing deficits may experience clinically relevant depression or anxiety, particularly if they perceive their hearing problems to be debilitating.

Consistent with prior studies, we found that subjective hearing handicap is a more potent correlation of psychological distress than objective hearing thresholds. HHIA scores had moderate correlations with both BDI-II (~0.45) and BAI (~0.38), whereas PTA’s correlations were weak (~0.2) and became non-significant in multivariate analysis. This indicates that how a person perceives and copes with their hearing loss is crucial in determining their emotional well-being. Two individuals with the same audiogram can have very different outcomes—one may adapt well with strong support systems, while another feels devastated and socially isolated [11]. Our multivariate results confirm that perceived hearing difficulties independently predict depression and anxiety severity, even when accounting for audiometric severity. This echoes findings by Huber et al. (2023) in cochlear implant candidates, where subjective hearing difficulties (on the APHAB questionnaire) correlated with BDI-II depression scores, while pure-tone measures did not [13]. Together, these data suggest that clinicians should not rely solely on audiograms to assess the impact of hearing loss; the patient’s self-reported hearing handicap and psychosocial challenges must also be considered [16]. Routine use of instruments like the HHIA, and direct questions about emotional and social effects of hearing loss, may help identify patients at risk for depression or anxiety who might otherwise be overlooked if one considers only the audiometric degree of loss.

Interestingly, neither age nor sex was associated with depression or anxiety severity in our cohort. In the general population, depression is often more common in women, and some studies have reported higher depression risk in older adults with hearing loss compared to middle-aged adults. Our sample (which deliberately excluded those over 65) did not show significant sex differences in BDI-II or BAI scores, nor any significant correlation between age (within 18–65) and mood. This could suggest that hearing loss imposes a fairly uniform psychological burden across adult subgroups. Even middle-aged adults in their 40s and 50s with hearing impairment can experience depression and anxiety at levels comparable to older adults with hearing loss. Past research has focused heavily on elderly or retired populations, but our results highlight that the middle-aged hearing-impaired population also warrants attention for mental health screening [17]. Some prior studies found no sex difference in depression among older hearing-impaired patients (e.g., Nilforoush et al. [12]), whereas others suggested women with hearing loss may be more prone to depression than men [18]. Our null finding for sex might reflect our sample size or the fact that we adjusted for multiple factors; it is also possible that in our sample women were slightly more likely to use hearing aids and have better social support, which might mitigate gender differences in mood outcomes.

The potential benefits of early hearing intervention for mitigating depression risk are an important consideration. Emerging evidence suggests that treating hearing loss can help reduce depressive symptoms in older adults [19]. For instance, a large cross-sectional study found that individuals with hearing loss who used hearing aids had significantly lower odds of moderate depression than those who did not use amplification (adjusted OR ~0.66) [20]. Likewise, a longitudinal study observed reductions in depressive symptoms within months of starting hearing aid use in previously untreated older adults [21]. In our sample, participants who used hearing aids tended to have slightly better mood scores (lower BDI-II/BAI) than non-users, although the difference was not statistically significant. This trend could be due to self-selection (those less depressed may be more consistent in using hearing aids), but it aligns with the idea that improving hearing can enhance social engagement and mood. The psychosocial benefits of audiological rehabilitation should be emphasized to patients. Audiologists and physicians can counsel patients that addressing hearing loss—through hearing aids, assistive devices, communication training, and environmental modifications—may help alleviate the feelings of frustration and isolation that contribute to depression [22]. It may also be prudent for audiology clinics to include brief mood screenings (e.g., PHQ-2/PHQ-9 for depression, GAD-7 for anxiety) as part of routine evaluations, in order to identify psychological distress early and refer patients for appropriate care.

Our results point to the importance of multidisciplinary management for patients with hearing loss. Treating the hearing deficit is crucial, but some individuals may also benefit from concurrent psychosocial support. Counseling or cognitive-behavioral therapy (CBT) techniques can help patients develop coping strategies for communication challenges and manage the emotional reactions to hearing loss (such as frustration, embarrassment, or low self-esteem). Support groups for people with hearing loss can reduce feelings of isolation and provide practical advice and encouragement from peers. Mental health professionals treating patients with depression or anxiety should remain alert to the possibility of undiagnosed hearing loss, especially in middle-aged and older adults. Simply asking patients about their hearing or referring them for a hearing evaluation when appropriate could improve overall treatment outcomes—unrecognized hearing impairment might otherwise hinder psychotherapy (for example, if the patient struggles to hear the therapist) and can contribute to social withdrawal. Some experts have even suggested that hearing evaluations be incorporated into the work-up for late-life depression.

Several limitations should be noted. First, the cross-sectional design limits our ability to infer causality; it is possible that mood disorders exacerbate perceived hearing problems rather than result from them (or both). Second, because inclusion was based on self-reported hearing trouble, there may be selection bias toward individuals who were already concerned about their hearing, potentially inflating the associations with mood. Third, our sample size (N = 100) was moderate and drawn from a combination of clinic visitors and community volunteers, which may limit generalizability. We also did not specifically assess tinnitus severity or cognitive function, which are factors that can influence mood and often co-occur with hearing loss.

Despite these limitations, our findings underscore the need for further research to clarify the causal pathways. Longitudinal studies could determine whether intervening to address hearing loss (for example, through hearing aid fitting or cochlear implants) leads to subsequent improvements in mood. Randomized controlled trials would be especially informative; for instance, assigning hearing-impaired individuals to receive early hearing intervention vs. delayed treatment and tracking depressive and anxious symptoms over time could provide high-quality evidence on the psychological benefits of audiological care. Future studies with larger and more diverse samples are also needed to explore whether certain subgroups (defined by age, sex, or coping resources) are particularly vulnerable to the mood effects of hearing loss.

## 5. Conclusions

In adults under age 65, hearing loss was strongly associated with depressive and anxious symptoms—especially when the individuals felt handicapped by their hearing loss. Subjective hearing handicap was a more robust predictor of psychological distress than audiometric hearing level. Clinicians should be attentive to mental health in patients with even mild-to-moderate hearing loss and consider early intervention. Conversely, mental health professionals should be aware of the potential impact of unrecognized hearing loss in patients presenting with depression or anxiety. While our study cannot prove causation, it highlights an important link between hearing and emotional well-being. Addressing hearing loss proactively—through amplification, communication strategies, and psychosocial support—may improve quality of life and should be investigated in future longitudinal research.

## Figures and Tables

**Table 1:** Participant characteristics and key psychometric measures.

Measure	Mean (SD) or %	Range
Age (years)	45.3 (11.2) 19–65	
Male, %	52% –	
Hearing loss severity (mild/mod-severe)	62%/38% –	
Uses hearing aid, %	41% –	
HHIA score	42.6 (22.8)	0–92
BDI-II score	16.8 (9.4)	0–42
BAI score	14.2 (8.7)	0–36

**Table 2:** Spearman correlation matrix for hearing loss metrics and mood scores.

Variables	Spearman ρ	*p*-Value
HHIA vs. BDI-II	0.45	<0.001
HHIA vs. BAI	0.38	<0.001
PTA vs. BDI-II	0.22	0.026
PTA vs. BAI	0.18	0.071

**Table 3:** Multivariate linear regression results for predictors of BDI-II depression score (N = 100). Note: Extended multivariate linear regression models including duration of hearing loss and hearing aid use can be found in Supplementary Table S1.

Predictor	Unstandardized β	Standardized β	95% CI	*p*-Value
HHIA score	0.37	0.37	0.22 to 0.52	<0.001
PTA (dB HL)	0.12	0.12	−0.05 to 0.30	0.16
Age	0.10	0.10	−0.09 to 0.29	0.30
Sex (0 = male, 1 = female)	−0.80	−0.08	−2.70 to 1.10	0.40

## Data Availability

The data presented in this study are not publicly available due to privacy considerations. Data may be available from the corresponding author on reasonable request.

## Supplementary Materials

The following supporting information can be downloaded at: https://www.mdpi.com/article/10.3390/healthcare13243320/s1, Table S1: Extended multivariate linear regression models including duration of hearing loss and hearing aid use.

## Abbreviations

BDI-II	Beck Depression Inventory–II
BAI	Beck Anxiety Inventory
HHIA	Hearing Handicap Inventory for Adults
PTA	Pure-Tone Average
dB HL	decibel Hearing Level
SNHL	sensorineural hearing loss
OR	odds ratio
CI	confidence interval
SD	standard deviation
IQR	interquartile range
PHQ	Patient Health Questionnaire
GAD	Generalized Anxiety Disorder (scale)
CBT	cognitive-behavioral therapy
APA	American Psychological Association (used for reference formatting)
CI (candidates)	cochlear implant (candidates)
SPSS	Statistical Package for the Social Sciences
IRB	Institutional Review Board
