# Supplementary material for: When Sound Fades: Depression and Anxiety in Adults with Hearing Loss—A Cross-Sectional Study
Source: Healthcare (Basel). 2025 Dec 18;13(24):3320. doi: 10.3390/healthcare13243320 (PMC12733058; doi:10.3390/healthcare13243320)
Supplement: Supplementary file 1 [file healthcare-13-03320-s001.zip › healthcare-4025452-supplementary.pdf]

Supplementary Table S1.
Extended multivariate linear regression models including duration of hearing loss and hearing aid use
Outcome 1: Depressive symptoms (BDI-II score)

Predictor	Unstandardized β	Standardized β	95% CI	p-value
HHIA score	0.35	0.36	0.21 – 0.50	<0.001
PTA (dB HL)	0.10	0.11	−0.07 – 0.27	0.24
Duration of hearing loss (years)	0.08	0.07	−0.12 – 0.28	0.42
Hearing aid use (yes/no)	−1.10	−0.09	−3.20 – 1.00	0.30
Age (years)	0.09	0.09	−0.10 – 0.28	0.34
Sex (female vs male)	−0.70	−0.07	−2.60 – 1.20	0.46

Model statistics:

- Adjusted $R^2 = 0.33$
- $F(6,93) = 9.8$, $p < 0.001$

Outcome 2: Anxiety symptoms (BAI score)

Predictor	Unstandardized β	Standardized β	95% CI	p-value
HHIA score	0.31	0.33	0.14 – 0.48	0.001
PTA (dB HL)	0.09	0.10	−0.08 – 0.26	0.29
Duration of hearing loss (years)	0.06	0.06	−0.15 – 0.27	0.58
Hearing aid use (yes/no)	−0.90	−0.08	−3.10 – 1.30	0.41
Age (years)	0.07	0.07	−0.11 – 0.25	0.44
Sex (female vs male)	−0.60	−0.06	−2.40 – 1.20	0.51

Model statistics:

- Adjusted $R^2 = 0.27$
- $F(6,93) = 6.7$, $p < 0.001$
